# In situ transplantation of adipose-derived stem cells via photoactivation improves glucose metabolism in obese mice

**DOI:** 10.1186/s13287-021-02494-4

**Published:** 2021-07-15

**Authors:** Luochen Zhu, Ziqian Feng, Xin Shu, Qian Gao, Jiaqi Wu, Zuoqin Du, Rong Li, Liqun Wang, Ni Chen, Yi Li, Mao Luo, Jianbo Wu

**Affiliations:** 1 Nantong Tumor Hospital (Tumor Hospital Affiliated to Nantong University), Nantong, Jiangsu People’s Republic of China; 2 Key Laboratory of Medical Electrophysiology of Ministry of Education, Collaborative Innovation Center for Prevention and Treatment of Cardiovascular Disease of Sichuan Province, Drug Discovery Research Center, Southwest Medical University, Luzhou, Sichuan People’s Republic of China; 3 Laboratory for Cardiovascular Pharmacology, Department of Pharmacology, School of Pharmacy, Southwest Medical University, Luzhou, Sichuan People’s Republic of China

**Keywords:** Photoactivation, Adipose tissue derived stem cells, Implantation, Insulin resistance, High-fat diet

## Abstract

**Background:**

Accumulating evidence suggests that enhanced adipose tissue macrophages (ATMs) are associated with metabolic disorders in obesity and type 2 diabetes. However, therapeutic persistence and reduced homing stem cell function following cell delivery remains a critical hurdle for the clinical translation of stem cells in current approaches.

**Methods:**

We demonstrate that the effect of a combined application of photoactivation and adipose-derived stem cells (ASCs) using transplantation into visceral epididymal adipose tissue (EAT) in obesity. Cultured ASCs were derived from subcutaneous white adipose tissue isolated from mice fed a normal diet (ND).

**Results:**

In diet-induced obesity, implantation of light-treated ASCs improved glucose tolerance and ameliorated systemic insulin resistance. Intriguingly, compared with non-light-treated ASCs, light-treated ASCs reduced monocyte infiltration and the levels of ATMs in EAT. Moreover, implantation of light-treated ASCs exerts more anti-inflammatory effects by suppressing M1 polarization and enhancing macrophage M2 polarization in EAT. Mass spectrometry revealed that light-treated human obese ASCs conditioned medium retained a more complete secretome with significant downregulation of pro-inflammatory cytokines and chemokines.

**Conclusions:**

These data suggest that the combined application of photoactivation and ASCs using transplantation into dysfunctional adipose tissue contribute to selective suppression of inflammatory responses and protection from insulin resistance in obesity and type 2 diabetes.

**Supplementary Information:**

The online version contains supplementary material available at 10.1186/s13287-021-02494-4.

## Introduction

During the progression of high-fat diet (HFD)-induced obesity, chronic low grade inflammation of visceral adipose tissue is closely linked to insulin resistance [1–4]. Enhanced adipose tissue macrophages (ATMs) appear to be a major contributing factor to the chronic inflammation associated with obesity and metabolic syndrome. Transplantation of subcutaneous adipose tissue into visceral depots improved systemic glucose intolerances by reducing circulating inflammatory cytokine levels in recipient HFD mice [5, 6]. In addition, inflammatory responses in adipose tissues increase free fatty acids, facilitating insulin resistance [7]. The therapeutic applications of mesenchymal stem cells (MSCs) have been investigated in experimental and preclinical studies. Several studies have shown that the intravenous infusion of MSCs has the potential to improve glucose homeostasis in diabetic animals by activating the insulin-signaling pathway and increasing the expression and membrane transposition of glucose transporters [8–10]. MSCs have been shown to modulate the microenvironment of pathological tissues contributing to repair and regeneration through secreting anti-inflammatory molecules [11–13]. We and others have demonstrated that fat tissue transplantation could reduce fat depot-selective ATM infiltration in obese mice [14, 15].

Accumulating studies have demonstrated the benefits of photoactivation as a promising and rapidly expanding physical approach in various pathologies, especially for reducing inflammatory responses and promoting tissue regeneration [12, 14, 16]. Using our specifically developed device with more refined wavelengths of light, we demonstrated that photoactivation shifted M1-to-M2 ATMs, and transplantation of the treated fat into the visceral adipose tissue improves glucose tolerance in obese mice [14]. Most recently, intravenous infusion of adipose-derived stem cells (ASCs) has shown that photoactivation prolonged functional blood flow perfusion and increased ASCs-derived EPC and neovascularization [12].

Despite the advantages of MSCs in repairing damaged tissue via cell replacement, however, challenges in the clinical application of MSCs, such as the low retention, survival of cells, the systemic administration route, and insufficient traffic to sites (homing) in vivo [17–19]. The development of novel delivery approaches provides a promising future for MSC-based therapies [20]. It is essential to note that adipose tissue has become an important therapeutic target. The biological functions of stem cells are communicated with the surrounding microenvironment known as the stem cell niche [21]. In fact, pharmacological modulation and remodeling of adipose tissues ameliorated systemic insulin resistance and enhanced insulin-dependent glucose uptake by suppressing fat depot-related ATM in obesity [13, 14].

In this study, we aimed to understand the biological effects of photoactivation. In particular, we explored the effect of a combined application of photoactivation and ASCs using transplantation into visceral epididymal adipose tissue (EAT) in obesity. Moreover, we performed in vivo fluorescently labeled mononuclear cell migration assays to determine the mechanism in explaining photoactivated ASCs-implanted differential ATMs infiltration in obesity. We report that the combined application of photoactivation and ASCs ameliorates hyperglycemia and insulin resistance in obese models via changing visceral ATM infiltration.

## Materials and methods

### Animals

Six- to 8-week-old C57BL/6J mice were obtained from the Chongqing Medical University Animal Center, Chongqing, China. All protocols for animal use were reviewed and approved by the Animal Care Committee of Southwest Medical University in accordance with Institutional Animal Care and Use Committee guidelines.

### HFD-fed mouse model

Eight-week-old male C57BL/6J mice were fed a high-fat diet (HFD) (TP2330055A; Research Diet, Trophic Animal Feed High-tech Co. Ltd, China) for 16 weeks as described previously [12]. Age-matched male mice that were fed a normal diet were used as controls. Blood samples were obtained from the tail vein and blood glucose levels were measured using an automatic glucometer (Accu-Chek; Roche Diagnostics, Mannheim, Germany). Body weight was monitored every seven days. Blood was collected and centrifuged at 1500*×g* for 10 min to measure fasting serum glucose.

### Culture of ASCs

Mouse adipose-derived stem cells (ASCs) were isolated from inguinal subcutaneous fat of C57BL/6 mice that were fed normal chow. The cells were cultured as described previously [12]. Briefly, subcutaneous adipose tissues were digested with collagenase type 1 (Sigma-Aldrich, St. Louis, MO, USA) in PBS (phosphate-buffered saline) by incubation in a shaker at 37 °C for 30 min. The digestion was terminated by the addition of 10% fetal bovine serum (FBS), and centrifuged at 1200 rpm for 5 min. Cells were suspended in complete medium made of DMEM/F12 (Dulbecco modified Eagle medium) medium supplemented with 10% FBS and 1% penicillin and streptomycin. Cells were cultured in 37 °C at 5% CO_2_ incubator. ASCs at passage 3–5 were used in all the experiments.

ASCs were identified by FACS as described previously [12] with fluorescence-conjugated anti-mouse antibodies, FITC Hamster Anti-Rat CD29 (BD Pharmingen; Cat.555005), Alexa Fluor 647 Rat anti-Mouse CD34 Clone RAM34 (RUO) (BD Pharmingen; Cat.560233), APC Rat Anti-Mouse CD90.2 Clone 53-2.1 (RUO) (BD Pharmingen; Cat.561974), APC anti-mouse CD105 (Biolegend; Cat.120413), PE anti-mouse CD31 (Biolegend; Cat.102407), or PE/Cy7 anti-mouse CD45 (Biolegend; Cat.103113), according to the manufacturer’s instructions. Flow cytometry was conducted on a Becton-Dickinson LSR I analyzer.

### Photoactivation

Cultured ASCs were transferred into a sterile syringe and subjected to light treatment for 30 min using a CellRegena Device (HarmonyRegena CO., Ltd, China) [12, 14]. The syringe was then placed into the device and rotationally activated by light-emitting diode (LED) light. This device integrates monochromatic lights of three different wavelengths, including 575–595 nm (5–20 mW), 630–635 nm or 660–670 nm (10–100 mW), and/or 510–540 nm (10–60 mW) of monochromatic light. A second set of cells was subjected to non-light treatment for 30 min as control group.

### ASCs transplantation

Adipose tissue was fractionated as previously described [15]. Briefly, male HFD for 16 weeks were used as recipients, and age-matched normal diet (ND)-fed mice were used as donors. ~ 2 × 10^6^ ASCs were mixed with 80 μL working solution of VitroGel (TheWell Bioscience, Inc.) and incubated at 37 °C for 20 min to form hydrogels, 20 μL DMEM medium were added into the total 100 μL of volume. The mixture of ASCs+ VitroGel was injected into the EAT in the right flanks of individual HFD-fed recipient mice, and equal volume of PBS was injected into EAT in the left flank as a vehicle.

### Monocyte isolation

Blood monocytes were isolated from ND-fed-C57BL/6J mice using EasySep Mouse Monocyte Enrichment kit (Stem Cell Technologies) as previously described [4]. Briefly, whole blood was collected by inferior venous cava, and erythrocytes were removed by hypotonic lysis (Pharmlyse, BD). Monocyte subsets were enriched with the EasySep mouse monocyte enrichment according to the manufacturer’s instructions.

### In vivo trafficking

The isolated blood monocytes were stained with CellTracker™ Green CMFDA (ThermoFisher) as previously described [4], and ~ 8 × 10^6^ cells were suspended in 0.2 mL PBS and injected into the tail vein of the each group of mice. Twelve hours after the injection, the ATMs were immediately isolated from EAT and analyzed in the flow cytometry.

### Flow cytometric analysis

FACS was performed as previously described [12]. Adipose stromal vascular cells were prepared from collagenase digested adipose tissue. The antibodies for surface staining were F4/80, Ly6C, CD11b, and CD11c (eBioscience, San Diego, CA) for macrophage analysis. The cells were analyzed using FACS Canto II (BD Biosciences). Numbers obtained were subsequently represented as the percentage of the highest subsets.

### Isolation of peritoneal macrophages

Normal chow-fed C57BL/6J mice were intraperitoneally injected with 1 mL of a 3% sterile thioglycollate solution (Difco Laboratories, Sparks, MD). After 4 days, peritoneal cells were harvested by washing the peritoneal cavity with PBS. Collected elicited-macrophages from peritoneum were counted by microscopy.

### Migration

To test peritoneal macrophage migration, ASCs (3 × 10^4^) were grown in the lower chambers of Transwell plates (8-μm pore size), and isolated peritoneal macrophages (1 × 10^4^) were added to the upper chambers. After 8-h incubation at 37 °C in a humidified chamber with 5% CO_2_ membranes were rinsed by dH_2_O and cells remaining in the upper chamber were removed by a cotton swab. Membranes were fixed and then stained with 0.5% crystal violet. Cells that migrated to the lower chamber were counted.

### Histological and immunohistochemistry

Mouse EAT was obtained and fixed in 4% (wt/vol) paraformaldehyde in PBS for 3h and subsequently transferred to 30% (wt/vol) sucrose overnight. The samples were then embedded in paraffin and serially sectioned (6 μm). Cross-sections were prepared for immunohistochemical staining overnight at 4 °C with a 1:100 dilution of a mouse mAb to Mac3 (M3/84; BD Biosciences), 1:400 dilution of a rat anti-mouse CD11c (GB11059, Servicebio), and 1:2000 dilution of a rat anti-mouse CD206 (GB13438, Servicebio). Immune complexes were detected with biotinylated secondary antibodies (BD Biosciences), HRP-conjugated streptavidin (Dako), and the peroxidase substrate diaminobenzidine (Dako). Images were captured using a microscope (Leica, Germany). Macrophage in EAT was quantitated by calculating the ratio of nuclei of Mac3-positive cells to total nuclei in 10 fields of 3 slides for each individual mice using 6 mice for each group [22]. The CD11c and CD206 staining level were validated quantitatively by image analysis assessing the pixel values on the digital slide images. In some experiments, cross-sections were stained with hematoxylin-eosin (HE). For quantification of adipocyte area, 5–6 fields per section were averaged and 6 mice per group were calculated. Image J software (NIH, USA) was used to measure adipocyte area and shown as the average adipocyte area (in μm^2^).

### Glucose uptake in ASCs

Confluent cultures of ASCs in 96-well plates were used to measure glucose uptake using Glucose Uptake-Glo assay kit (Promega Corporation, USA). Briefly, cells were starved for 4 h in DMEM 25 mM Glucose with 0.25% BSA. Cells were incubated with 100 nM insulin for 20 min in KRH buffer (137 nM NaCl, 4.8mMKCl, 1.2 mM KH2PO4, 1.2 mM MgSO4, 2.5 mM CaCl2, 16 mM HEPES) supplemented with 0.2% BSA at 37 °C; then, cells were incubated with 0.2 mM 2-deoxy-D-glucose diluted in KRH buffer and incubated for additional 10 min at 37 °C. Cells were lysed in RIPA buffer and 2-deoxy-glucose-6-phosphate was measured using a luminometer.

### Quantitative real-time PCR

EAT were collected 10 weeks post-transplantation and total RNA was extracted using TRIzol reagent (Invitrogen, Carlsbad, CA, USA). RNA samples were pre-treated with deoxyribonuclease I (Invitrogen Life Technologies, Carlsbad, CA, USA), and a SuperScript kit (Invitrogen Life Technologies, Carlsbad, CA, USA) was used to synthesize cDNA according to the manufacturer’s recommendations. qRT-PCR was analyzed using miScript SYBR Green PCR Kits (Qiagen). Levels of macrophage polarization and oxidative stress markers mRNAs were determined by ABI PRISM 7700 cycler (Applied Biosystems, Foster City, CA). Each sample was analyzed in duplicate with ribosomal 18S RNA as an internal control. All fold changes in gene expression were determined using the 2^−ΔΔCT^ method. The values are presented as the mean ± SEM. All primers are listed in Table S3.

### Immunoblotting

ASCs lysates were prepared, and equal amounts of protein were subjected to SDS-PAGE and transferred to polyvinylidene difluoride membranes by electroblotting. After blocking, the membranes were incubated with antibodies directed against phospho-Akt (Ser473) and total AKT (Cell signaling). Secondary antibody was horseradish-peroxidase (HRP)-conjugated goat IgG raised against IgG (Santa Cruz Biotechnology). Blots were developed with ECL substrate (Pierce).

### Glucose and insulin tolerance testing

A glucose tolerance test (GTT) and an insulin tolerance test (ITT) were performed by following intraperitoneal (IP) injections of D-glucose (Roth, Karlsruhe, Germany) (2 g of glucose/kg body mass) and insulin (0.75 U insulin/kg body mass) after a 4-h fast, respectively. Blood samples were then obtained from the tail vein, and the blood glucose levels were measured at 0, 30, 60, and 120 min using a One Touch® Vita® glucometer (Zug, Switzerland).

### Plasma parameters

Plasma IL-6, insulin, and leptin concentrations were measured by ELISA kits (ThermoFisher Scientific, USA). Plasma FFA and glycerol levels were measured by specific assay kits (Abcam).

### hASCs conditioned media harvest and Mass spectrometry analysis

Human normal or obese adipose-derived stem cells (hASCs, Catalogs #: 10HU-001; 10HU-232) were obtained from iXCells Biotechnologies (San Diego, CA, USA) and were grown in BulletKit TM Medium (LONZA, Walkersville Inc.). hASCs (passage 3 to 4) were cultured until 80% confluence. Collected cells were subjected to light treatment for 30 min using a CellRegena Device and cultured with serum free medium for 72 h. The second set of cells was subjected to non-light treatment for 30 min as a control group. CM was prepared from three different biological replicates of normal hASCs and obese hASCs in the presence or absence of light treatment. For hASCs secretomes harvest and mass spectrometry analysis were performed as described previously [12]. Briefly, an aliquot of 5 μl peptide extract was analyzed by LC-MS/MS on a UHPLC system (1290, Agilent Technologies) with a UPLC HSS T3 column (1.8 μm, 2.1 × 100 mm, Waters) coupled to a Q Exactive mass spectrometer (Orbitrap MS, Thermo). LC-MS/MS data were processed with acquisition software (Xcalibur 4.0.27, Thermo) using default settings depending on preselected criteria. The cellular function was defined using the Ingenuity Pathway Analysis (Qiagen). Enriched pathways were visualized by Cytoscape (3.2.1) software. Evaluation of overrepresented GO biological processes of proteins was performed using Panther (www.pantherdb.org) and a Fisher’s exact with FDR multiple test correction.

### Statistical analysis

Data are presented as the mean ± SEM of triplicate experiments. The significance of the differences among groups was analyzed by one-way analysis of variance with a post hoc test to determine group differences in the study parameters. All analyses were performed with SPSS software (version 24.0 for Windows; Armonk, NY, USA), and a level of *P* < 0.05 was defined as indicative of statistical significance.

## Results

### Transplantation of photoactivated ASCs improves glucose homeostasis

**Fig. 1:**
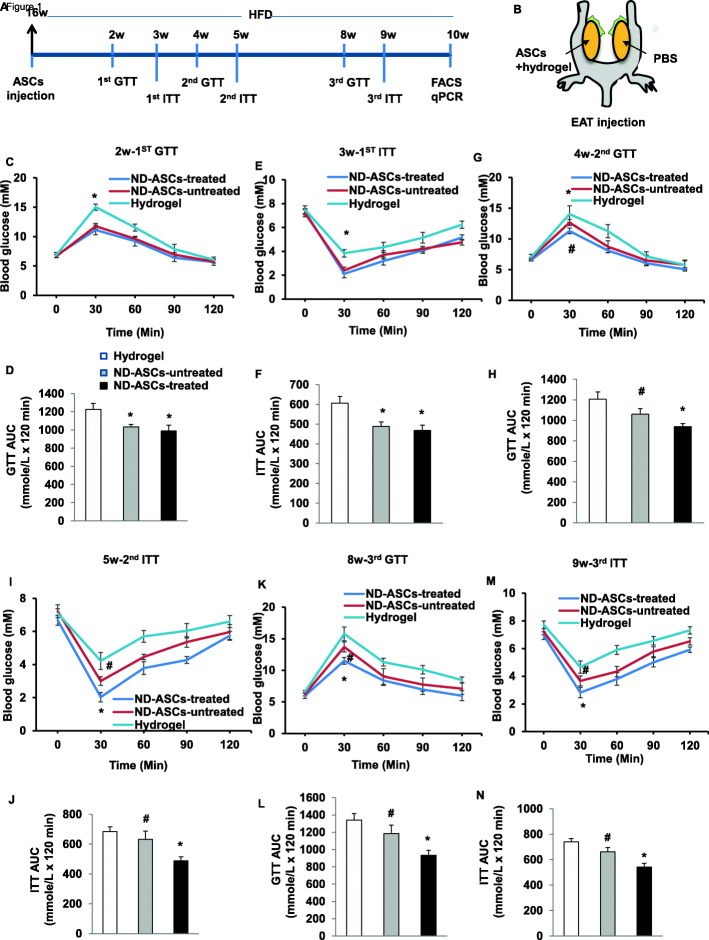
Transplantation of photoactivated ASCs improves glucose homeostasis. **A** Schematic timeline of ASCs transplantation model. Mice were fed HFD for 16 weeks, followed by transplantation of light-treated ASCs+hydrogel, non-light-treated ASCs+hydrogel, or hydrogel alone into right EAT (EAT-R) for 10 weeks. **B** An experimental strategy to examine the effects of ASCs in vivo. In two EATs from each HFD recipient mouse, one EAT-R was locally injected with either light-treated ASCs+hydrogel, non-light-treated ASCs+hydrogel, or hydrogel alone, whereas the controlateral EAT (EAT-L) was injected with vehicle (PBS). **C**, **E**, **G** Blood glucose levels and **D**, **F**, **H** areas under the curve during the GTT in HFD mice that received light-treated ASCs+hydrogel, non-light-treated ASCs+hydrogel, or hydrogel alone at 2, 4, and 8 weeks after implantation, respectively. n = 6–8 per group; *p < 0.05 vs. hydrogel alone; **p < 0.05 vs. non-light-treated ASCs+hydrogel and hydrogel alone; and ^#^p > 0.05 vs. hydrogel alone. **I**, **K**, **M** Blood glucose levels and **J**, **L**, **N** areas under the curve during the ITT in HFD mice that received light-treated ASCs+hydrogel, non-light-treated ASCs+hydrogel, or hydrogel alone at 3, 5, and 8 weeks after implantation, respectively. n = 6–8 per group; *p < 0.05 vs. non-light-treated ASCs+hydrogel and hydrogel alone; and ^#^p > 0.05 vs. hydrogel alone. EAT, epididymal adipose tissue

To investigate whether transplantation of photoactivated-ASCs could improve glucose tolerance and insulin resistance, we fed mice high-fat chow for 16 weeks, which produced obesity and hyperglycemia (Supplementary Figure S1A-C). Following the schematic timeline (Fig. 1A) and strategy (Fig. 1B) provided in the experimental model, either light-treated ASCs+hydrogel, the same number of light-untreated ASCs+hydrogel, or hydrogel alone was injected into EAT on the right, while equal volume of vehicle (PBS) was injected into EAT on the left of 16 weeks-HFD recipient mice, and HFD loading was then performed for 10 weeks. The mouse body weights were not significantly different between the three groups. We evaluated the effect of ASCs transplantation on glucose tolerance and insulin sensitivity. At 2–3 weeks after cell implantation, HFD mice receiving light-treated ASCs+hydrogel or light-untreated ASCs+hydrogel showed faster glucose disposal during the GTT compared with hydrogel alone (Fig. 1C, D). Mice receiving light-treated ASCs+hydrogel or light-untreated ASCs+hydrogel also showed increased insulin sensitivity with marked reductions in blood glucose levels than hydrogel alone during the ITT (Fig. 1E, F). However, there were no differences in GTT and ITT in mice receiving light-treated ASCs+hydrogel than non-light-treated ASCs+hydrogel group.

To further determine the duration of the protective effect of ASCs therapy, we examined GTT and ITT again at 4–9 weeks after cell implantation. HFD mice receiving all two kinds of ASCs showed still faster glucose clearance and increased insulin sensitivity. Notably, the light-treated ASCs group showed significantly lower glucose levels than the non-light-treated ASCs+hydrogel and the hydrogel groups at 4 and 8 weeks for GTT (Fig. 1G, H, K, L), and 5 and 9 weeks for ITT (Fig. 1I, J, M, N). In contrast, the non-light-treated group showed a lightly decreased tendency compared with hydrogel group. These data suggest that implantation of ASCs to visceral adipose tissues improved glucose clearance and insulin sensitivity in vivo. It is important to note that photoactivation exhibits a prolonged therapeutic effect on adipose-related glucose homeostasis.

### Transplantation of photoactivated ASCs decreases the accumulation of adipose tissue macrophages

**Fig. 2:**
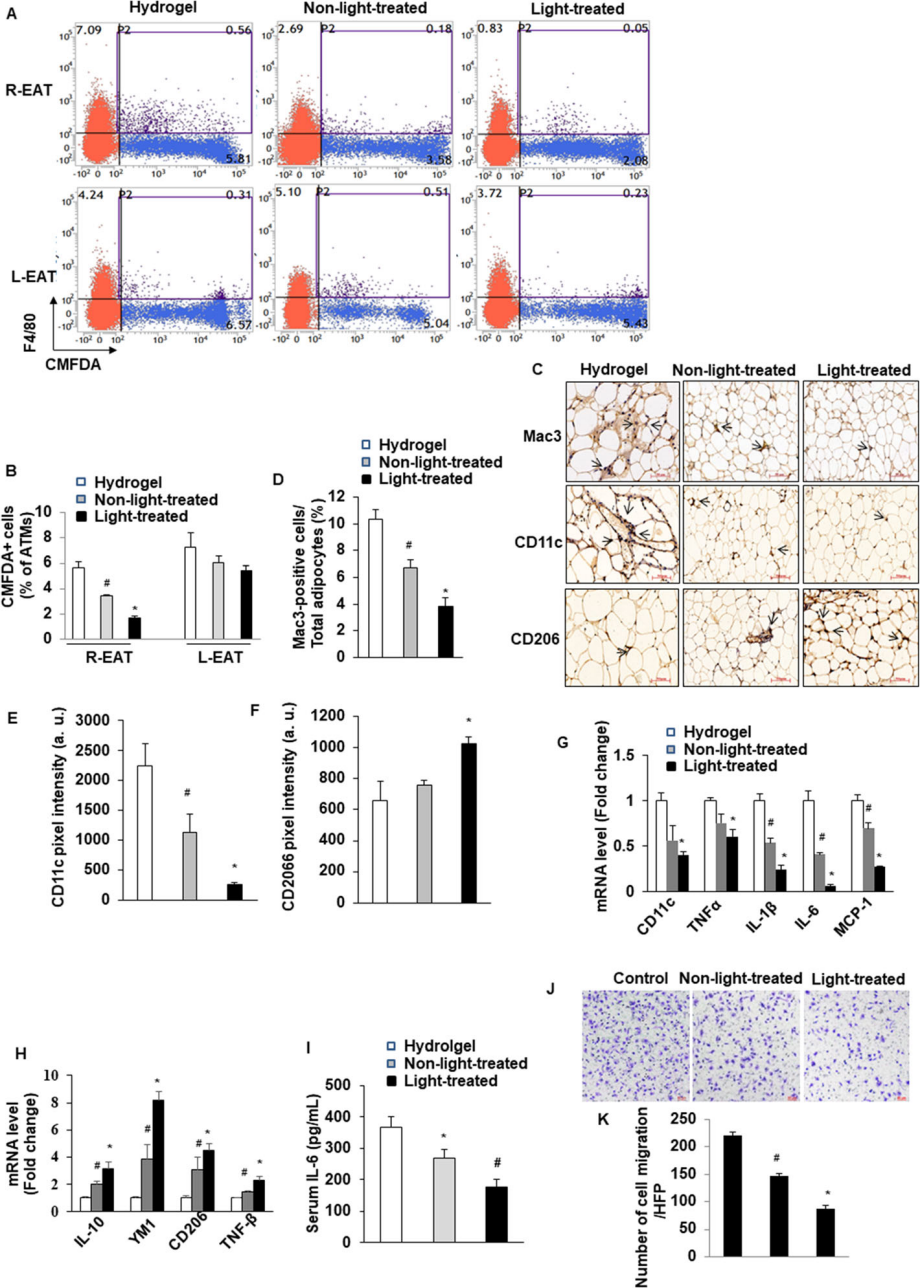
Transplantation of photoactivated ASCs decreases the accumulation of adipose tissue macrophages. **A**, **B** Quantitation by flow cytometry of the proportion of CMFDA+ ATMs in the SVF of EAT from HFD mice that received light-treated ASCs+hydrogel, non-light-treated ASCs+hydrogel, or hydrogel alone. n = 6–8 per group; *p < 0.05 vs. non-light-treated ASCs+hydrogel and hydrogel alone; and ^#^p < 0.05 vs. hydrogel alone. Data are mean ± SEM. **C**–**F** Immunohistochemical detection of Mac3 (**D**), CD11c, and CD206 in EAT of from HFD mice that received light-treated ASCs+hydrogel, non-light-treated ASCs+hydrogel, or hydrogel alone. n = 6–8 per group; *p < 0.05 vs. non-light-treated ASCs+hydrogel and hydrogel alone; and ^#^p < 0.05 vs. hydrogel alone. Data are mean ± SEM. Macrophages are stained brown, indicated as the ratio of Mac3-positive cells to total cells. Quantification of the pixel intensity of CD11c (**E**) and CD206 (**F**) indicating the degree of pro- or anti-inflammatory polarization of macrophage in EAT. Magnification, × 200. Scale bars, 50 μm. a.u.: arbitrary unites. **G**, **H** Quantitative RT-PCR analysis of total RNA isolated from EAT of HFD recipient mice for IL-6, IL-1β, TNF-α, MCP-1, CD11c, IL-10, YM1, TNF-β, and CD206 mRNAs. Data were normalized by the amount of 18s mRNA and expressed relative to the corresponding hydrogel alone. n = 6–8 per group. *p < 0.05 vs. non-light-treated ASCs+hydrogel and hydrogel alone; and ^#^p< 0.05 vs. hydrogel alone. Data are mean ± SEM. **I** Serum IL-6 level was measured by ELISA (n = 6/each group); *p < 0.05 vs. non-light-treated ASCs+hydrogel and hydrogel alone; and ^#^p > 0.05 vs. hydrogel alone. **J** The isolated HFD-C57/6J mice-derived peritoneal macrophages were added to upper chambers. ASCs from either light-treated or no-light-treated were placed in the lower chambers. Cells were fixed after 8 h and stained with crystal violet. Migrated cells were counted. Representative images of cell migration are shown. **K** Quantitative assessment of triplicate cell migration experiments was performed. Data shown are mean ± SEM. *p < 0.05 vs. control (medium without ASCs) and non-light-treated; ^#^p < 0.05 vs. control

We hypothesized that transplantation of ASCs into AT would exacerbate inflammatory infiltration. Indeed, our previous ex vivo study found that light-treated white adipose tissue (WAT) has shifted M1 macrophage phenotype into M2 in HFD mice [14]. To confirm and extend the observation, we directly measured monocyte migration into adipose tissue using an in vivo macrophage tracking technique. With this approach, isolated circulating monocytes were obtained from ND-fed-C57BL/6J donor mice and labeled with CMFDA-Green dye. The labeled monocytes were then injected into recipient HFD-fed mice with the treatment of hydrogel alone, non-light-treated ASCs+hydrogel, and light-treated ASCs+hydrogel, respectively. We performed FACS analysis of stromal vascular fraction (SVF) of EAT. The results showed that the percentage of CMFDA+ ATMs was markedly lower in light-treated ASCs+hydrogel than in non-light-treated ASCs+hydrogel and hydrogel alone groups (Fig. 2A, B). However, there were no significant differences in the percentage of CMFDA+ ATMs in left EAT between groups, which were only injected with PBS (Fig. 2A, B).

We further evaluated the number of infiltrated macrophages in adipose tissue by immunohistochemical analysis with Mac3 antibody. We counted the degree of macrophage infiltration by calculating the ratio of infiltrated macrophages to total cells in EAT. The result showed that Mac3-positive macrophage infiltration was decreased significantly in both light-treated ASCs mice and non-light-treated ASCs mice compared with hydrogel alone mice (Fig. 2C, D). We next evaluated the effect of implanted ASCs on the polarization state of macrophages in EAT. Sections were stained for CD11c, a pro-inflammatory M1 marker of ATMs. CD11c staining intensity was predominantly lower in light-treated ASCs mice than in non-light-treated ASCs and hydrogel alone mice (Fig. 2C, E), whereas light-treated ASCs resulted in high expression of the M2 markers CD206 in EAT (Fig. 2C, F). Furthermore, consistent with these findings, we found significantly decreased expression of a variety of pro-inflammatory genes by qRT-PCR. As shown in Fig. 2G, the mRNA levels of pro-inflammatory genes, including IL-6, IL-1β, TNF-α, MCP-1, and CD11c, were slightly decreased in the non-light-treated ASCs, compared with hydrogel alone. Moreover, the implantation of light-treated ASCs was significantly downregulated these M1 macrophage pro-inflammatory genes. Similarly, the light-treated ASCs upregulated the mRNA levels of IL-10, YM1, TNF-β, and CD206 (Fig. 2H). However, there were no differences in PBS-injected EAT between the three groups (Supplementary Figure S2). Furthermore, after 10 weeks of ASCs implantation, the serum level of IL-6 was determined by enzyme-linked immunosorbent assay. The serum IL-6 level was significantly lower in light-treated ASCs mice than in non-light-treated ASCs and hydrogel alone mice (Fig. 2I).

Next, we observed that effect of light-treated ASCs on the macrophage migration. Macrophages were isolated from normal chow-fed C57BL/6J mice. As shown in Fig. 2J and K, macrophage migration towards either light-treated or non-light-treated ASCs was significantly impaired compared with the control group. Interestingly, light-treated ASCs exhibited a significantly more reduction in migration compared with non-light-treated ASCs. These results indicate that photoactivation of ASCs exerts more anti-inflammatory effects by suppressing M1 polarization and enhancing macrophage M2 polarization in adipose tissue.

### Transplantation of photoactivated ASCs improves metabolic parameters and morphological change in obese mice

**Fig. 3:**
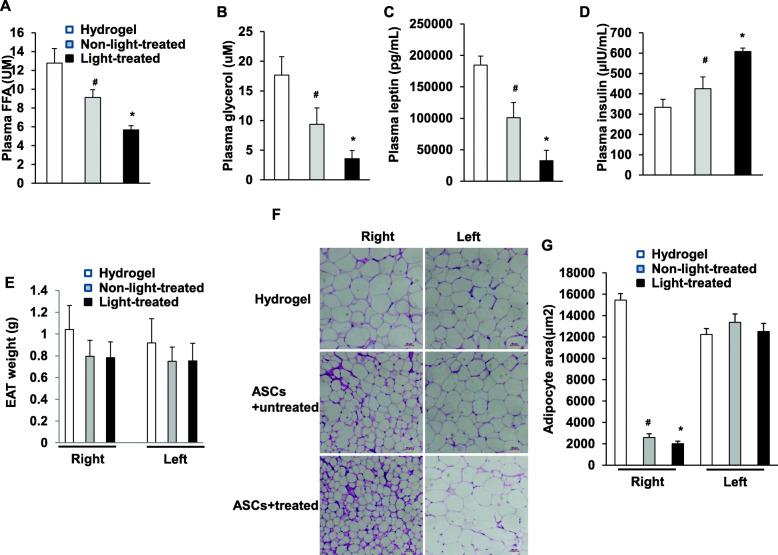
Transplantation of photoactivated ASCs improves metabolic parameters and morphological change in obese mice. HFD mice received light-treated ASCs+hydrogel, non-light-treated ASCs+hydrogel, or hydrogel alone, in which **A** plasma FFA, **B** plasma glycerol, **C** plasma leptin, **D** plasma insulin, and **E** EAT weight were measured (n = 6–8 per group); *p < 0.05 vs. non-light-treated ASCs+hydrogel and hydrogel alone; and ^#^p < 0.05 vs. hydrogel alone. Data are mean ± SEM. **F** EAT sections were stained by hematoxylin. Representative histological images from HFD mice that received light-treated ASCs+hydrogel, non-light-treated ASCs+hydrogel, or hydrogel alone. **G** The area of adipocyte size is presented as graphs. Data shown are mean ± SEM. n = 6–8 per group; *p < 0.05 vs. non-light-treated ASCs+hydrogel and hydrogel alone; and ^#^p > 0.05 vs. hydrogel alone. EAT, epididymal adipose tissue

We measured metabolic parameters of these HFD mice that received implantation of ASCs at 10 weeks. Plasma free fatty acid, glycerol, and leptin concentrations were significantly lower in mice that received light-treated ASCs than in mice that received non-light-treated and hydrogel alone (Fig. 3A–C). As the main effect of insulin on adipose tissue is lipolysis inhibition. Plasma insulin concentration was significantly higher in mice that received light-treated ASCs than in mice that received non-light-treated and hydrogel alone (Fig. 3D). The body weight of HFD mice was not changed significantly by implantation of ASCs (Supplementary Figure S1D). Either light-treated or non-light-treated ASCs caused a slight decrease in the weight of EAT, although not statistically significant (Fig. 3E). The area of epididymal adipocytes decreased significantly in mice that received light-treated ASCs and non-light-treated ASCs compared with mice that received hydrogel alone (Fig. 3F, G). In contrast, there is no significant difference in the weight of controlateral EAT between the three groups.

### Photoactivation directly improves cellular insulin resistance

**Fig. 4:**
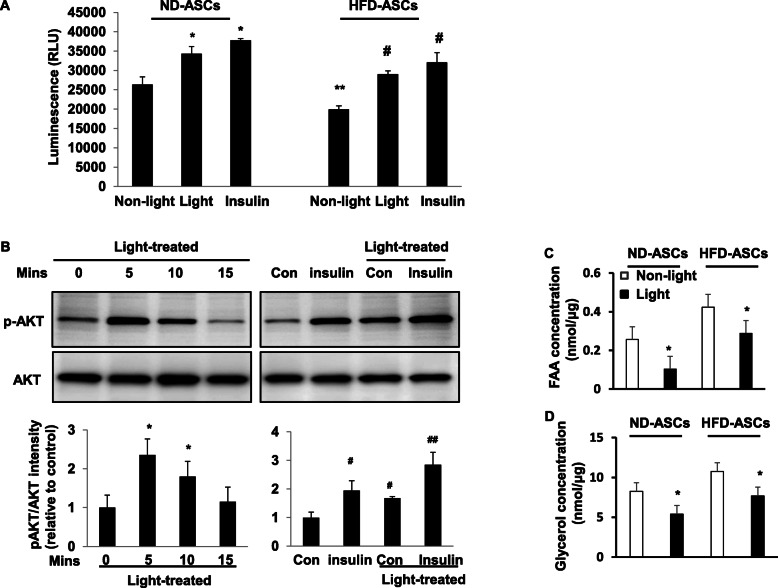
Photoactivation directly improves cellular insulin resistance. **A** Effects of 30 min of light treatment on glucose uptake in ND-ASCs or HFD-ASCs. Cells were stimulated with 100 nM insulin for 20 min as a positive control. *p < 0.05 vs. non-light-treated ND-ASCs; **p < 0.05 vs. non-light-treated ND-ASCs; and ^#^p < 0.05 vs. non-light-treated HFD-ASCs. Data are mean ± SEM (n = 3 independent treatments). RLU, relative luminescence units. **B** ND-ASCs were cultured, and light treatment in the stimulation with insulin (50 Mm) for 30 min as indicated. Cell lysates were prepared and subjected to Western blotting to detect phosphorylated and total AKT. All graphs correspond to the blots above them and represent densitometric analyses of 3 independent experiments. *P < 0.05 vs. non-light-treated; ^#^p < 0.05 vs. non-light-treated; and ^##^p < 0.05 vs. light-treated. **C**, **D** Levels of FFA and glycerol in cultured ND-ASCs supernatant were measured after 30 min of light treatment. *P < 0.05 vs. non-light-treated

Given that implantation of photoactivated ASCs could improve glucose tolerance and insulin sensitivity in obesity, we asked whether photoactivation could directly act on glucose uptake to induce insulin-AKT signaling. To assess this, we studied the effects of photoactivation treatment on glucose uptake from normal diet (ND) and HFD-ASCs. As shown in Fig. 4A, compared with non-light treatment in ND-ASCs, 30 min of light treatment promotes glucose uptake similar to insulin. The ND-ASCs showed a higher basal level of glucose uptake than that in HFD-ASCs.

To further understand the mechanism of photoactivation-induced glucose uptake in ASCs, the insulin-AKT signaling was studied in cultured cells.

The phospho-Akt (Ser473) in cultured ND-ASCs was significantly increased after light treatment at 5 and 10 mins (Fig. 4B). Furthermore, the stimulation with the low-insulin concentration (50 mM) for 20 mins significantly increased AKT phosphorylation in the non-light-treated ASCs. It showed a robust increase in AKT phosphorylation when applied in the light-treated ASCs than in non-light-treated ASCs (Fig. 4B).

We further determined the levels of FFA and glycerol in cultured ND-ASCs supernatant after light treatment. As shown in Fig. 4C and D, compared with non-light treatment in ND-ASCs, 30 min of light treatment significantly reduces the concentrations of FAA and glycerol. Similar effect was observed in HFD-ASCs from light treatment.

### Proteomic analysis of cultured hASCs secretome under photoactivation

**Fig. 5:**
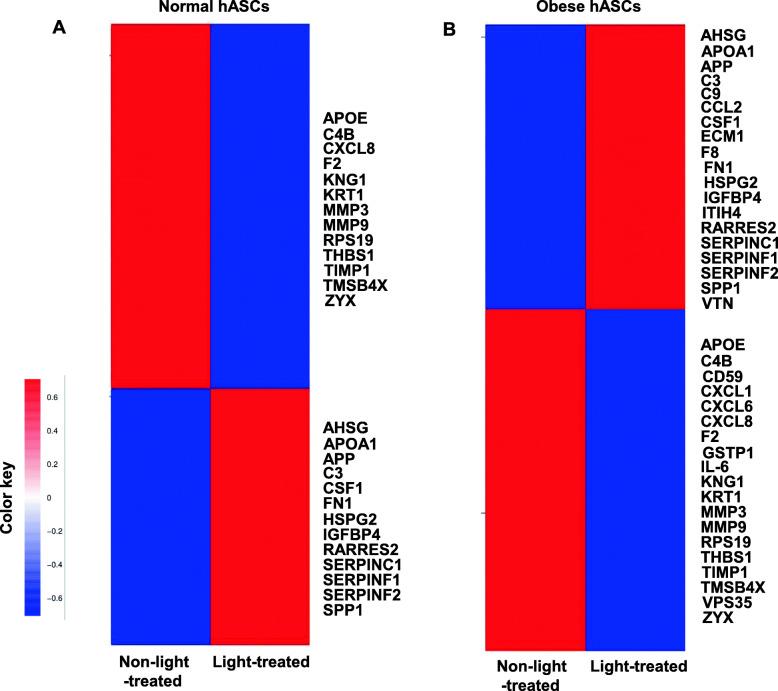
Proteomic analysis of cultured hASCs secretome. Heatmap indicating protein distribution in light-treated and non-light-treated hASCs, as determined by quantitative proteomics. Hierarchical clustering is shown in normal hASCs (**A**), and obese hASCs (**B**), clusters of positive (red; upregulated) and negative (green; downregulated) correlations showed the log 2-fold expression changes

The proteome of human ASCs (hASCs) CM (conditional medium) was analyzed based on the protein intensities measured by LC/MS-MS. We investigated the secreted protein profiles of normal and obese human ASCs. A total of 266 proteins in the normal hASCs CM and 361 proteins in the obese hASCs CM were identified in the comparative analyses (Table S1-2), including 28 of normal ASCs-derived and 38 of obese ASCs-derived secreted proteins were involved in the inflammatory response (Table 1). Heatmap demonstrated the differential protein expression profile between light-treated CM and non-light-treated CM (Fig. 5A, B). The numbers of downregulated secretome in the normal ASCs CM-and obese ASCs CM-light-treated groups were 21 and 19, respectively. The pro-inflammatory cytokines and chemokines, such as CXCL1, CXCL6, CXCL8, and matrix metalloproteinases, including MMP3 and MMP9, were significantly downregulated by the light treatment. IL-6 was only identified in obese hASCs CM and significantly downregulated by the light treatment compared with normal hASCs CM.

**Table 1:** Enriched biological processes based on regulated inflammatory proteins

Cell type	Genes involved	p value
Normal hASCs	RPS19/APP/SERPINF2/C4B/THBS1/CXCL8/RARRES2/F2/AHSG/SPP1/APOE/SDC1/CSF1/MMP3/APOA1/A2M/TIMP1/C3/SERPINC1/KNG1/FN1/KRT1/MMP9/IGFBP4/SERPINF1/TMSB4X/HSPG2/ZYX	0.0001
Obese hASCs	RPS19/APP/SERPINF2/C4B/F8/CD59/THBS1/IL6/ITIH4/CXCL8/RARRES2/F2/AHSG/SPP1/CXCL6/APOE/CSF1/MMP3/VTN/APOA1/TIMP1/CCL2/C3/SERPINC1/KNG1/C9/FN1/KRT1/GSTP1/CXCL1/MMP9/IGFBP4/SERPINF1/TMSB4X/HSPG2/ZYX/ECM1/VPS35	0.0001

## Discussion

This study developed a novel and direct approach for improving glucose homeostasis by implanting normal chow ASCs to visceral adipose tissue in HFD-induced obese mice, which reduced adipose tissue monocyte/macrophage infiltration, converted M1 macrophage polarization to M2, and downregulated pro-inflammatory cytokines and chemokines through photoactivation. This procedure provides a therapeutic strategy for improving insulin sensitivity and glucose homeostasis by implanting ASCs to adipose tissue, thereby enhancing the metabolic activity of the hosts via photoactivation.

Photoactivation treatments are widely used in various biomedical and clinical applications. Recently, light treatment has been applied in conjunction with stem cell proliferation and proangiogenic effects in animal experiments [12, 14, 16]. Accumulating evidence has shown the infusion of MSCs to improve d*iet*-induced *obesity* and *metabolic disorders by* decreasing blood glucose levels and promoting regeneration of pancreatic islet of diabetic animals [9–13]. Stem cells are able to incorporate in the microenvironment of injured tissues contributing to tissue repair and regeneration through the secretion of anti-inflammatory molecules, cytokines, and chemokines with immunomodulatory properties [10, 16]. It is well established that visceral adipose tissue ATMs are positively correlated with insulin resistance and metabolic diseases in obesity [23, 24]. However, their efficacy in treating type 2 diabetes remains elusive though venous infusion in the most study. Using direct implantation of cells offers a possible solution to the adipose tissue homing challenge, while ASCs could provide direct anti-inflammatory effects, improving systemic glucose hemostasis. Therefore, improvement of cell status and setting environment for their in vivo application may be a potential strategy for enhancing the function of ASCs.

The strategies can be involved in two parts: suppressing the number of infiltrated monocytes/macrophages or altering their functionality within the adipose tissue in obesity. We found that infiltration of exogenous ATM was significantly lower in mice receiving light-treated ASCs than in mice receiving non-light-treated ASCs. Indeed, our findings reveal that photoactivation of ASCs decreases macrophage migration in vitro. Our results indicated that light-treated human obese ASCs secretome might have a more prone anti-inflammatory profile when compared with non-light-treated ASCs. Consistent with such results in vitro, both EAT IL-6 mRNA and serum IL-6 level were significantly lower in light-treated ASCs mice than in non-light-treated ASCs and hydrogel alone mice. IL-6 is secreted from adipose tissue and is known to be elevated in obesity and insulin resistance. Although the precise mechanism underlying the anti-inflammatory properties of light-treated ASCs was not addressed in our current study, the paracrine secretion has been shown more critical in tissue repair and regeneration [24, 25]. Previous studies have shown that ASCs-derived exosomes drove M2 macrophage polarization in WAT, which further promoted adipose tissue remodeling [26]. We found that photoactivation of ASCs exerts cellular insulin sensitizing effects, at least in part by modulating the AKT phosphorylation, and correlates with decreased the release of FFA and glycerol. Similarly, the implantation of light-treated ASCs resulted in a striking decrease in plasma FFA and glycerol levels.

In obese and type 2 diabetic subjects, adipose tissue has been shown to contain increased numbers of M1 polarization macrophages, a major source of pro-inflammatory cytokines [27–29]. Our previous study has been shown that light treatment selectively decreased the expression of markers for M1 macrophages in subcutaneous WAT [14]. In the present study, we showed that the implantation of light-treated ASCs promoted M1-to-M2 macrophage polarization. M2-related anti-inflammatory cytokine such as IL-10 in EAT might act as a compensatory mechanism involving the insulin-signaling pathway to maintain glucose homeostasis in obesity.

Inflammation in the adipose tissue may inhibit ASCs differentiation into preadipocytes and caused impaired adipocyte hyperplasia and insulin-signaling pathway [29, 30]. ASCs are multipotent cells and exhibit the capacity of self-renewal functions with regenerative properties. Our recent study demonstrated that photoactivation increases ASCs proliferation in vitro [12]. Although implantation of ASCs did not appear to affect the total mass of EATs, histological analysis revealed that either light-treated or non-light-treated ASCs did cause a decrease in the area of adipocytes and an increase in the number of smaller adipocytes. The photoactivation could be attributed to accelerations in ASCs self-renewal, which eventually resulted in dysfunctional adipose tissue remodeling and improvements in metabolic homeostasis.

There are several limitations to our study. Notably, we have only demonstrated that the implantation of light-treated ASCs into visceral epididymal adipose tissue improves systemic insulin sensitivity using mouse obese models. However, compelling evidence suggests that subcutaneous adipose tissues are less susceptible to ATMs accumulation. Studies on time are needed to test its efficacy and safety. Future studies will be required to define the functional phenotypic markers of macrophage activation, and the signaling pathway of modulating the intracellular molecules by photoactivation. The differentiation of ASCs and implantation of ASCs alone in adipose tissue were not analyzed in our study. Future experiments into the mode of action involving photoactivation and enhancing local cell differentiation and survival may improve the therapeutic efficacy of this method.

## Conclusions

In conclusion, this study provides an essential clue to understanding the suppression of ATMs in improving glucose homeostasis and insulin resistance in obesity. We developed photoactivation of ASCs as a simple and effective therapeutic strategy that targets ATMs infiltration in obesity. Taken together, our studies propose that the combined application of photoactivation and ASCs would be an attractive approach to treating obesity-induced metabolic disorders.

## Supplementary Information

**Additional file 1: Table S1.** The normal hASCs CM (conditional medium) were identified in the comparative analyses.

**Additional file 2: Table S2.** The obese hASCs CM were identified in the comparative analyses.

**Additional file 3: Table S3.** Primers used in qPCR.

**Additional file 4: Supplementary Figure S1.** High-fat diet induces obesity, hyperglycemia, and body weights in mice. Mice were fed high-fat diet (HFD) or normal diet (ND) for 16 weeks, after which (A) GTT, (B) ITT, (C) body weight, and (D) body weight post-transplantation were measured (n=6/group); *P<0.05 vs. ND group. **Supplementary Figure S2.** Quantitative RT-PCR analysis of total RNA isolated from L-EAT of HFD recipient mice for IL-6, IL-1β, TNF-α, MCP-1, and CD11c, IL-10, YM1, TNF-β, and CD206 mRNAs. Data were normalized by the amount of 18s mRNA and expressed relative to the corresponding hydrogel alone. n = 6-8 per group.

## Data Availability

The data generated or analyzed during this study are included in this article, or if absent are available from the corresponding author upon reasonable request.

## Abbreviations

ASCs: Adipose-derived stem cells
ATMs: Adipose tissue macrophages
CM: Conditional medium
EAT: Epididymal adipose tissue
GTT: Glucose tolerance test
HFD: High-fat diet
ITT: Insulin tolerance test
MSCs: Mesenchymal stem cells
ND: Normal diet
WAT: White adipose tissue
