# Supplementary material for: In situ transplantation of adipose-derived stem cells via photoactivation improves glucose metabolism in obese mice
Source: Stem Cell Res Ther. 2021 Jul 15;12:408. doi: 10.1186/s13287-021-02494-4 (PMC8281693; doi:10.1186/s13287-021-02494-4)
Supplement: Supplementary file 3 — Additional file 3: Table S3. Primers used in qPCR. [file 13287_2021_2494_MOESM3_ESM.docx]

**Primers used in qPCR**

| **Gene** |  | **Forward** | **Reverse** |
| --- | --- | --- | --- |
| CD11c |  | CACTCAGTGACTGCCCAAAA | CCTCAAGACAGGACATCGCT |
| IL-1β |  | ACTACAGGCTCCGAGATGAACAAC | CCCAAGGCCACAGGTATTTT |
| CCR2 |  | GCAAGTTCAGCTGCCTGCAA | ATGCCGTGGATGAACTGAGGTAA |
| CD206 |  | CATGGATGTTGATGGCTACTGGAG | GTCTGTTCTGACTCTGGACACTGG |
| MCP-1 |  | ATGCAGGTCCCTGTCATG | GTTCACTGTCACACTGGTCA |
| IL-6 |  | CACATGTTCTCTGGGAAATCG | TTGTATCTCTGGAAGTTTCAGATTGTT |
| TNF-α |  | ACGGCATGGATCTCAAAGAC | AGATAGCAAATCGGCTGACG |
| Ly6C |  | ACTGTGCCTGCAACCTTGTC | CACACAGTAGGGCCACAAGA |
| IL-10 |  | TGTCAAATTCATTCATGGCCT | ATCGATTTCTCCCCTGTGAA |
| Ym1 |  | AGAGTGCTGATCTCAATGTGG | GGGCACCAATTCCAGTCTTAG |
| TGF-β1 |  | TGCTAATGGTGGACCGCAA | CACTGCTTCCCGAATGTCTGA |
| Actin |  | AAGAGCTATGAGCTGCCTGA | TACGGATGTCAACGTCACAC |
