# Supplementary material for: In situ transplantation of adipose-derived stem cells via photoactivation improves glucose metabolism in obese mice
Source: Stem Cell Res Ther. 2021 Jul 15;12:408. doi: 10.1186/s13287-021-02494-4 (PMC8281693; doi:10.1186/s13287-021-02494-4)

**Supplementary Figure S1.** High-fat diet induces obesity, hyperglycemia, and body weights in mice. Mice were fed high-fat diet (HFD) or normal diet (ND) for 16 weeks, after which (A) GTT, (B) ITT, (C) body weight, and (D) body weight post-transplantation were measured (n=6/group); *P<0.05 vs. ND group.


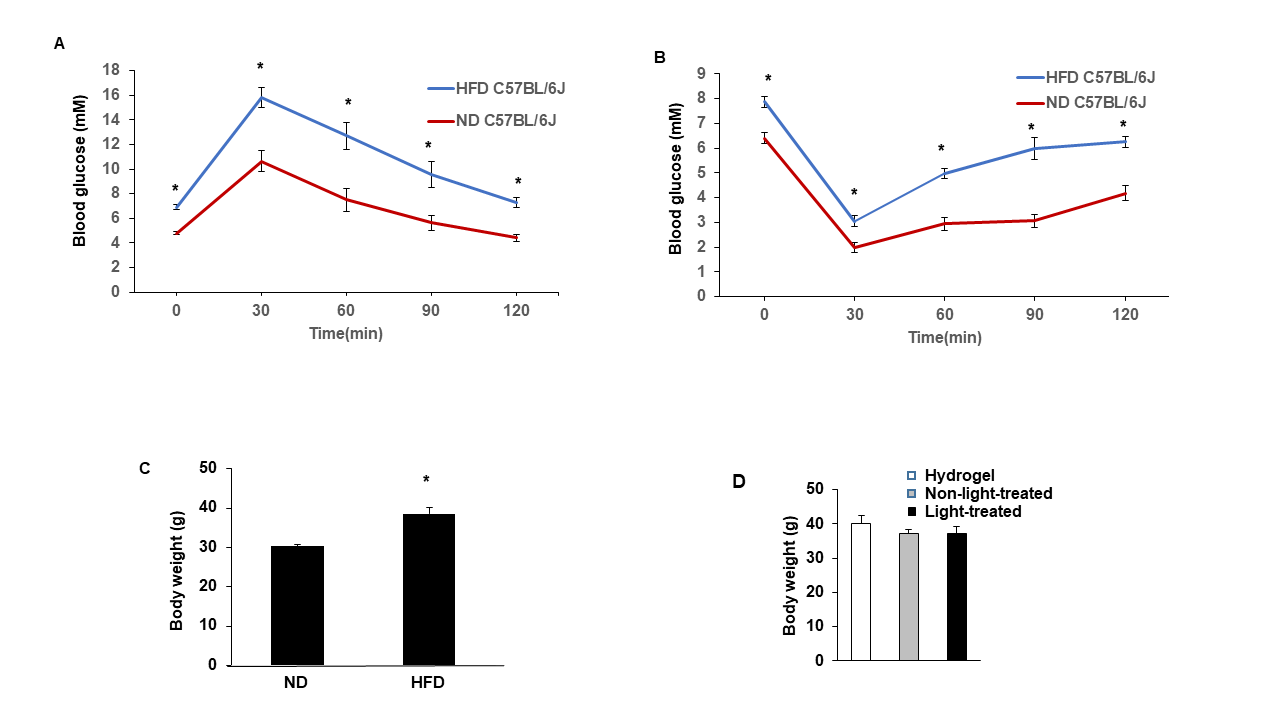


**Supplementary Figure S2.** Quantitative RT-PCR analysis of total RNA isolated from L-EAT of HFD recipient mice for IL-6, IL-1β, TNF-α, MCP-1, and CD11c, IL-10, YM1, TNF-β, and CD206 mRNAs. Data were normalized by the amount of 18s mRNA and expressed relative to the corresponding hydrogel alone. n = 6-8 per group.


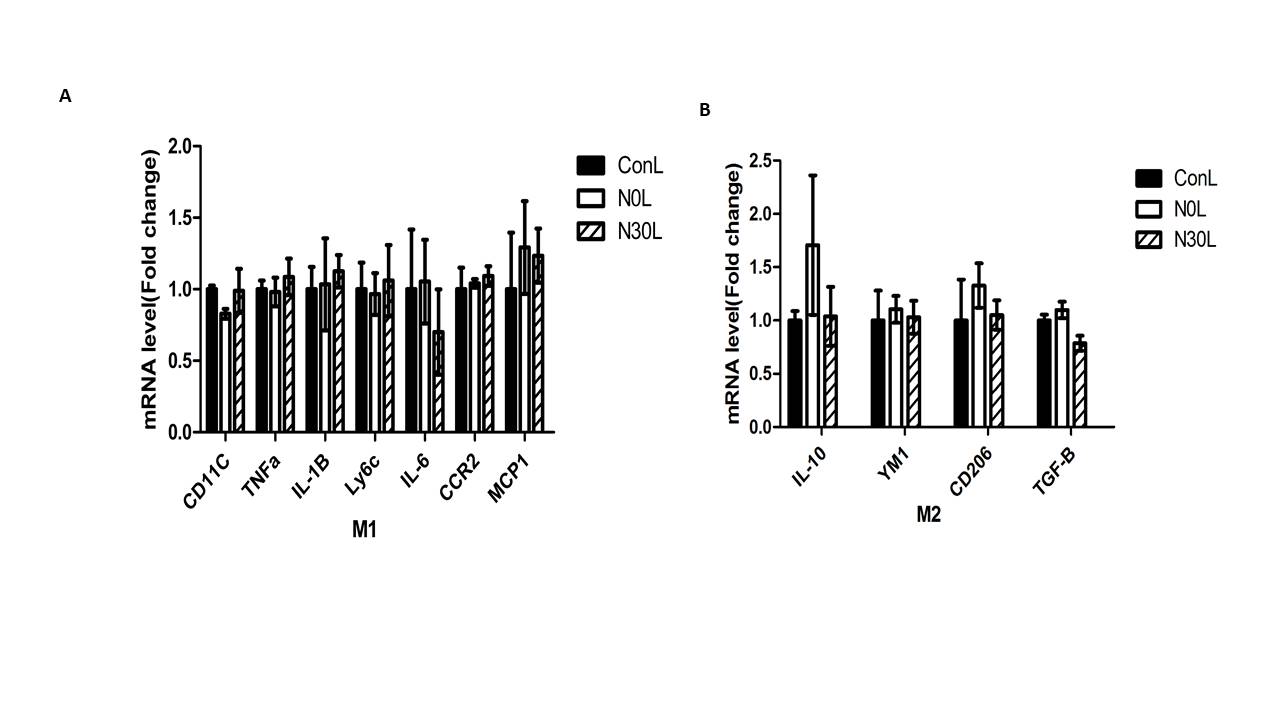

Supplement: Supplementary file 4 — Additional file 4: Supplementary Figure S1. High-fat diet induces obesity, hyperglycemia, and body weights in mice. Mice were fed high-fat diet (HFD) or normal diet (ND) for 16 weeks, after which (A) GTT, (B) ITT, (C) body weight, and (D) body weight post-transplantation were measured (n=6/group); *P<0.05 vs. ND group. Supplementary Figure S2. Quantitative RT-PCR analysis of total RNA isolated from L-EAT of HFD recipient mice for IL-6, IL-1β, TNF-α, MCP-1, and CD11c, IL-10, YM1, TNF-β, and CD206 mRNAs. Data were normalized by the amount of 18s mRNA and expressed relative to the corresponding hydrogel alone. n = 6-8 per group. [file 13287_2021_2494_MOESM4_ESM.docx]
